# Isoforskolin Alleviates AECOPD by Improving Pulmonary Function and Attenuating Inflammation Which Involves Downregulation of Th17/IL-17A and NF-κB/NLRP3

**DOI:** 10.3389/fphar.2021.721273

**Published:** 2021-07-30

**Authors:** Chuang Xiao, Sha Cheng, Runfeng Li, Yutao Wang, Deyou Zeng, Haiming Jiang, Yaping Liang, Rong Huang, Hanxiao Pan, Xiao Wu, Yan Fang, Chen Chen, Xian Li, Rongping Zhang, Xinhua Wang, Zifeng Yang, Weimin Yang

**Affiliations:** ^1^ School of Pharmaceutical Science and Yunnan Key Laboratory of Pharmacology for Natural Products, Kunming Medical University, Kunming, China; ^2^ State Key Laboratory of Respiratory Disease, National Clinical Research Center for Respiratory Disease, Guangzhou Institute of Respiratory Health, The First Affiliated Hospital of Guangzhou Medical University, Guangzhou, China; ^3^ School of Traditional Chinese Medicine, Yunnan University of Chinese Medicine, Kunming, China

**Keywords:** AECOPD, isoforskolin, pulmonary function, Th17 cell, NF-κB, NLRP3

## Abstract

Chronic obstructive pulmonary disease (COPD), a major cause of morbidity and mortality worldwide, is widely considered to be related to cigarette smoke (CS), and viral infections trigger acute exacerbation of COPD (AECOPD). Isoforskolin (ISOF) is a bioactive component from the plant *Coleus forskohlii*, native to Yunnan in China. It has been demonstrated that ISOF has anti-inflammatory effect on acute lung injury animal models. In the present study, we investigated the efficacy and mechanism of ISOF for the prevention and treatment of AECOPD. Mice were exposed to CS for 18 weeks and then infected with influenza virus A/Puerto Rico/8/34 (H1N1). ISOF (0.5, 2 mg/kg) was intragastrically administered once a day after 8 weeks of exposure to cigarette smoke when the body weight and lung function of model mice declined significantly. The viral load, pulmonary function, lung morphology, Th17 cells, and inflammatory cytokines in lung tissues were evaluated. The expression of nuclear factor κB (NF-κB) and NOD-like receptor pyrin domain–containing protein 3 (NLRP3) inflammasome pathways were detected. The results showed that ISOF treatment reduced the viral load in the lung homogenate, decreased the lung index of model mice, and lung pathological injuries were alleviated. ISOF also improved the pulmonary function with increased FEV0.1/FVC and decreased Rn and Rrs. The levels of inflammatory mediators (TNF-α, IL-1β, IL-6, IL-17A, MCP-1, MIG, IP-10, and CRP) in the lung homogenate were reduced after ISOF treatment. ISOF decreased the proportion of Th17 cells in the lung tissues by the flow cytometry test, and the protein expression levels of RORγt and p-STAT3 were also decreased. Furthermore, ISOF significantly inhibited the activation of NF-κB signaling and NLRP3 inflammasome in the lung tissues of model mice. In conclusion, ISOF alleviates AECOPD by improving pulmonary function and attenuating inflammation via the downregulation of proinflammatory cytokines, Th17/IL-17 A, and NF-κB/NLRP3 pathways.

## Introduction

Chronic obstructive pulmonary disease (COPD) is a common preventable and treatable disease that is characterized by persistent respiratory symptoms and airflow obstruction, and it is currently the third largest cause of human mortality worldwide (Rabe and Watz, 2017; Collaborators, 2020; Whittaker Brown and Braman, 2020). Acute exacerbation of COPD (AECOPD), defined as “an acute worsening of respiratory symptoms that result in additional therapy,” is the main cause of high hospitalization rates and mortality in COPD patients (Collaborators, 2020; Li et al., 2020; Whittaker Brown and Braman, 2020). The major risk factor of COPD is cigarette smoke (CS), and viral or bacterial respiratory infections result in COPD exacerbations with increased airway inflammation and mucus secretion (Whittaker Brown and Braman, 2020; MacLeod et al., 2021).

According to the guidelines of Global Initiative for Chronic Obstructive Lung Disease (GOLD), long-acting β2-adrenoceptor agonists (LABAs), long-acting muscarinic antagonists (LAMAs), and inhaled corticosteroids (ICSs) were recommended to treat COPD patients with severe symptoms and/or high risk exacerbation history (Collaborators, 2020). Additionally, phosphodiesterase-4 inhibitor, antibiotics, or antiviral drugs could be beneficial for specific subsets of AECOPD patients (Li et al., 2020; Whittaker Brown and Braman, 2020; MacLeod et al., 2021). Despite the progress in symptom treatment and prevention of exacerbations, it is still challenging to develop novel treatments to ameliorate disease progression or affect mortality (Rabe and Watz, 2017; Whittaker Brown and Braman, 2020).

The pathogenesis of COPD includes oxidative stress, protease–antiprotease imbalance, inflammatory cells and mediators, cellular senescence, mitochondrial dysfunction, and metabolic dysregulation (Zhang et al., 2018; Hikichi et al., 2019). Inflammation cells including neutrophils, macrophages, dendritic cells, T cells, and B cells are all involved in COPD pathogenesis (Ni and Dong, 2018; Zhang et al., 2018). Cigarette smoke and other toxic substances can trigger the release of inflammatory cytokines (TNF-α, IL-1β, IL-6, IL-8, and IL-17A) in the lung tissue, which would recruit inflammatory cells, increase inflammatory responses, and aggravate the pulmonary injury (Zhang et al., 2018; Wang et al., 2020). Th17 cells, a subtype of activated CD4^+^ T cells, could promote the systemic inflammation of COPD through the secretion of IL-17A (Miossec and Kolls, 2012; Ponce-Gallegos et al., 2017; Ni and Dong, 2018).

The nuclear factor-κB (NF-κB) also plays an important role in the systemic inflammation of COPD patients (Wang et al., 2020). Bacterial or viral infection activates the NF-κB signaling pathway, which could regulate the gene expression of cytokines and chemokines, further promoting the disease development (Schuliga, 2015; Wang et al., 2020). Recent studies suggest that inflammasome plays a role in the pathogenesis of COPD, especially in infection-related exacerbations of COPD (Kim et al., 2015; Faner et al., 2016; Wang et al., 2018). NOD-like receptor NLRP3 can recognize a variety of pathogen-associated molecular patterns (PAMPs) and danger-associated molecular patterns (DAMPs) to activate the NLRP3 inflammasome (Broz and Dixit, 2016; Evavold and Kagan, 2018; Zhao and Zhao, 2020). Generally, the activation of NLRP3 inflammasome requires the priming step and the activation step. In the priming step, activation of the NF-κB signaling pathway facilitates the expression of NLRP3, pro-caspase-1, pro-IL-1β, and pro-IL-18 (Broz and Dixit, 2016; Zhao and Zhao, 2020). In the activation step, NLRP3 interacts with apoptosis-associated speck-like protein (ASC) and recruits pro-caspase-1 to form the protein complex called the NLRP3 inflammasome, leading to the activation of caspase-1 and, subsequently, the secretion of IL-1β and IL-18 (Broz and Dixit, 2016; Zhao and Zhao, 2020). The activation of NLRP3 inflammasome can result in downstream inflammatory responses and the initiation of adaptive immunity (Pinkerton et al., 2017; Evavold and Kagan, 2018).

Isoforskolin (ISOF) is a natural product from the plant *Coleus forskohlii* native to Yunnan in China which can activate adenylyl cyclase (AC) and increase the intracellular cAMP (Yang et al., 2001; Yang et al., 2011). Given that cAMP plays a critical role in the regulation of immune and inflammatory function, elevating intracellular cAMP level is considered beneficial to the treatment of pulmonary diseases (Maurice et al., 2014; Zuo et al., 2019; Schmidt et al., 2020). Previously, we showed that ISOF attenuated lipopolysaccharide- (LPS-) induced acute lung injury *in vivo* (Yang et al., 2011) and downregulated proinflammatory cytokines *in vitro* through the TLR4/MyD88/NF-κB signaling pathway (Du et al., 2019). Moreover, ISOF has been reported to inhibit airway remodeling and inflammation in a rat asthma model induced by ovalbumin (Liang et al., 2017). We also found that ISOF could alleviate pathological changes in an elastase- or CS-induced COPD model (application patent: ZL201410083987.8). However, the effect of ISOF on AECOPD has not been reported and further investigation is needed.

Thus, in this study, we aimed to explore the efficacy and mechanisms of ISOF on AECOPD. We established an AECOPD mice model induced by long-term CS exposure and H1N1 infection. The effect of ISOF on pulmonary function, lung morphology, and proinflammatory cytokines in the lung tissues of AECOPD model mice were evaluated, and the potential molecular mechanisms involving Th17/IL-17A axis and NF-κB/NLRP3 signaling pathway were clarified.

## Materials and Methods

### Reagents and Antibodies

ISOF (PubChem CID: 9549169, Figure 1A, purity >99.9%) was synthesized by Basilea Pharmaceutica (Jiangsu, China). Oseltamivir phosphate (OSE) was from Meilunbio (Dalian, China). Prednisone acetate (PDN) and vecuronium bromide were purchased from Zhejiang Xianju Pharmaceutical (Zhejiang, China). Cigarettes (Hongqi Canal® Filter tip cigarette, smoke of each cigarette containing 11 mg tar, 0.7 mg nicotine, and 13 mg carbon monoxide) were obtained from Henan Tobacco Industry (Zhengzhou, China). The Bio-Plex Pro-Mouse Cytokine panel was from Bio-Rad (California, United States). Mouse CXCL9/MIG ELISA kit, mouse CXCL10/IP-10 ELISA kit, and mouse C-Reactive Protein/CRP ELISA kit were from MultiSciences (Hangzhou, China). MACS Lung Dissociation Kit (Cat. #130-095-927) was from Miltenyi Biotec (California, United States). Leukocyte activation cocktail (Cat. #550583), Cytofix/Cytoperm solution kit (Cat. #554714), lysis buffer (Cat. #555899), fixable viability stain 620 (Cat. #564996), FITC anti-mouse CD4 (Cat. #553046), and PE anti-mouse IL-17A (Cat. #561020) were purchased from BD Biosciences (New Jersey, United States). Anti-mouse CD16/32 (Cat. #101320) and PE/Cy7 anti-mouse CD3 antibody (Cat. #100220) were purchased from BioLegend (California, United States). PerCP-eFluor 710 anti-mouse CD8 (Cat. #46-0081-80) was purchased from eBioscience (California, United States). Mouse STAT3 antibody (Cat. #sc-8019), mouse phospho-STAT3 antibody (Cat. #sc-8059), and mouse RORγt antibody (Cat. #sc-293150) were from Santa Cruz Biotechnology (California, United States). Mouse IκBα antibody (Cat. #4814), rabbit phospho-IκBα antibody (Cat. #2859), rabbit NF-κB p65 antibody (Cat. #8242), rabbit phospho-NF-κB p65 antibody (Cat. #3033), rabbit NLRP3 antibody (Cat. #15101), rabbit ASC antibody (Cat. #67824), rabbit IL-1β antibody (Cat. #12426), rabbit Caspase-1 antibody (Cat. #24232), and rabbit GAPDH antibody (Cat. #2118) were from Cell Signaling Technology (Massachusetts, United States).

**FIGURE 1:**
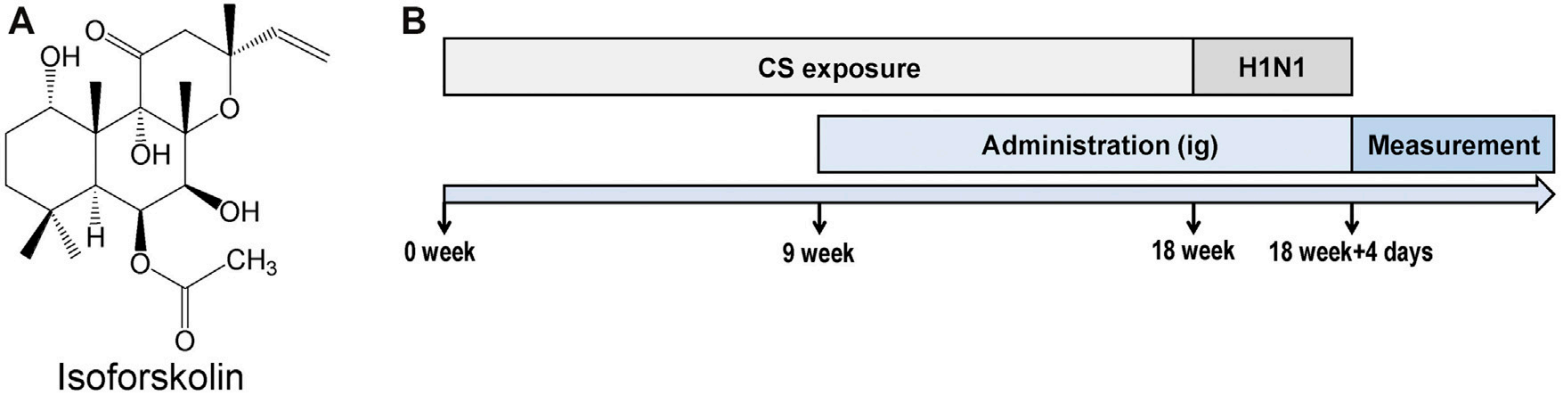
Experimental design. **(A)** Structure of isofoskolin. **(B)** Experimental flow chart. Mice were exposed to cigarette smoke once a day for 18 weeks and then intranasally challenged with H1N1 virus, and measurements were performed on the 4th day after infection. Mice were orally administrated with ISOF (0.5 or 2.0 mg/kg) or PDN (5 mg/kg) once daily after 8 weeks of exposure to cigarette smoke. OSE (60 mg/kg) was administered intragastrically before H1N1 infection.

### Acute Exacerbation of COPD Model Mice and Treatment

All animal care and experimental protocols were approved by the Animal Experimental Ethical Committee of Kunming Medical University, and all animals received humane care in compliance with the National Institutes of Health guidelines. C57BL/6 J male mice (20 ± 2 g) were purchased from Charles River Laboratories (Beijing, China). After acclimatization for a week, mice were randomly divided into the following groups: control, model, OSE (60 mg/kg), PDN (5 mg/kg), ISOF 0.5 mg/kg, and ISOF 2.0 mg/kg groups (*n* = 12 per group).

The experimental flow chart is shown in Figure 1B. Animals were exposed to room air or cigarette smoke once a day for 18 weeks (1 cigarette each mouse) in an oral and nasal exposure system (Beijing Huironghe Technology, China). The relevant parameters of exposure were as follows: dilution flow of 10 L/min, air extraction flow of 13 L/min, oxygen concentration of 20 ± 0.5%, and air humidity of 60 ± 5%. The cigarette suction parameters included the following: suction time of 2 s, time interval of 2 s, suction frequency of 10 s, and suction flow of 35 ml. Mice were orally treated with PDN (5 mg/kg) or ISOF (0.5 or 2.0 mg/kg) once daily after 8 weeks of exposure to cigarette smoke. OSE (60 mg/kg) was administered intragastrically before H1N1 (influenza virus A/Puerto Rico/8/34, ATCC, United States) infection. Mice in the control group and model group were orally treated with 0.9% saline solution (80%) and PEG400 (20%). The median lethal dose (LD_50_) of H1N1 was determined in another study (Wu et al., 2021). After 18 weeks of exposure to cigarette smoke, mice were anesthetized with 2.5% isoflurane and intranasally challenged with 50 μl of H1N1 (2 × LD_50_) or PBS. 4 days after infection, mice underwent euthanasia after lung function measurement. The left lung tissues were used for histopathology or flow cytometry analysis, and the right lung tissues were homogenized or stored at −80°C. The viral titer in the lung homogenate was determined by a 50% tissue culture infective dose (TCID_50_) assay as previously described (Zhou et al., 2020).

### Pulmonary Function Examination

Pulmonary function in conscious mice was assessed every two weeks by whole body plethysmography (EMKA Technologies, Canada). The respiratory parameters obtained using this technique include expiratory time (Te), peak expiratory flow (PEF), expiratory flow at 50% tidal volume (EF50), and maximum minute ventilation (MV).

Invasive lung function was measured with a FlexiVent system (SCIREQ, Montreal, Canada). A plastic cannula was inserted into mouse tracheas and connected to the FlexiVent system. Vecuronium bromide (6 mg/kg) was injected intraperitoneally to maintain muscle relaxation. Then, mechanical ventilation was initiated to measure forced expiratory volume in 0.1 s (FEV0.1), forced vital capacity (FVC), FEV0.1/FVC, Newtonian resistance (Rn), respiratory resistance (Rrs), and tissue damping (G). Each maneuver was performed at least three times, and the data obtained were analyzed using the FlexiVent software (SCIREQ, Montreal, Canada).

### Lung Histopathology and Immunohistochemistry

Mouse lung tissues were embedded in 4% paraformaldehyde, sectioned at 3–5 μm thicknesses, and stained with hematoxylin and eosin (H&E). The histopathological lesions and changes were observed under a light microscope (Olympus, Tokyo, Japan). The severity of pulmonary inflammation was assessed by a semiquantitative analysis as described previously (Triantaphyllopoulos et al., 2011). For immunohistochemistry staining, lung tissue paraffin sections were stained by antibodies against NLRP3 according to the manufacturer’s instructions. Density of yellow and brown reflects the level of target protein. Negative control slides with omission of primary antibody were included and did not show any immunoreaction. Representative images of IHC staining were obtained using an optical microscope (Olympus, Tokyo, Japan).

### Detection of Proinflammatory Cytokines

Lung tissues from each mouse were homogenized in PBS by SCIENTZ-48 (SCIENTZ, China) and then centrifuged at 12,000 rpm for 10 min to obtain supernatants. The cytokines TNF-α, IL-1β, IL-6, IL-17A, and CCL2/MCP-1 were measured by the Bio-Plex Pro Mouse Cytokine panel using the Bio-Plex 200 Multiplex Immunoassay system (Bio-Rad, California, United States) according to the manufacturer’s protocol. The concentration of CXCL9/MIG, CXCL10/IP-10, or C-reactive protein (CRP) was measured by a specific enzyme-linked immunosorbent assay (ELISA) kit. The procedure of ELISA was performed according to the manufacturer’s instructions.

### Flow Cytometry Analysis

Lung tissues of mice were dissociated by the MACS Lung Dissociation kit according to the instructions. In brief, the lung tissues of mice were transferred into the gentleMACS C tube and the enzyme mix was added. Then, the C tube was attached to the gentleMACS dissociator (Miltenyi Biotec, California, United States) and the program was started to run. The mixtures were filtered through a 70 μm cell strainer to obtain the cell suspension, and red blood cells were lysed with lysis buffer. Cells were resuspended with 1 ml RPMI-1640 cell culture media in 24-well plates, and 2 μl/ml of leukocyte-activation cocktail was added. Then, they were mixed briefly and incubated for 5 h in 5% CO_2_ at 37°C.

Cell preparations were stained with fixable viability stain 620 in PBS. After centrifugation, cell suspension in FACS buffer was preincubated with anti-mouse CD16/32 (1 µg) at 4°C for 5 min and then stained with PE/Cy7 anti-mouse CD3, FITC anti-mouse CD4, and PerCP-eFluor 710 anti-mouse CD8 antibodies at 4°C for 30 min. After surface staining, cells were resuspended in a fixation and permeabilization solution according to the manufacturer’s instructions and then stained with PE anti-mouse IL-17A. The cells were washed, resuspended, and detected using a flow cytometer (PARTEC CyFlow Space, Germany).

### Western Blot Analysis

Lung tissues from each mouse were homogenized in lysis buffer (RIPA: Cocktail: PMSF: Phosstop = 0.657: 0.143: 0.1: 0.1) to extract total protein. The protein concentration was determined with a BCA protein assay kit (Beyotime, China). Total protein (30 μg) was separated by 10% sodium dodecyl sulfate–polyacrylamide gel electrophoresis and transferred to polyvinylidene difluoride (PVDF) membranes and then incubated with blocking buffer (Beyotime, China) at room temperature for 2 h. The antibody of STAT3, p-STAT3, RORγt, IκBα, *p*-IκBα, p65, p-p65, NLRP3, ASC, IL-1β, caspase-1, or GAPDH was incubated at 4°C with the membrane overnight. After washing three times, the membrane was incubated with horseradish peroxidase–conjugated anti-rabbit IgG or anti-mouse IgG for 2 h at room temperature. Then, the membrane was washed three times and detected with a DAB horseradish peroxidase color development kit (Beyotime, China). The density of the protein bands in the membrane was quantified by Scion Image 4.0.2 (Informer Technologies, United States).

### Statistical Analysis

Data were expressed as means ± SEM. Statistical analysis was performed by using SigmaStat 3.5 software (Systat Software, United States). Multiple group comparisons were performed using one-way analysis of variance (ANOVA), followed by Fisher’s least significant difference (LSD) test to determine significant differences between different groups. Kruskal–Wallis ANOVA on ranks was used if there was no homogeneity of data variance. A *p* value of less than 0.05 was considered statistically significant.

## Results

### Isoforskolin Improved Pulmonary Function in Acute Exacerbation of COPD Model Mice

**FIGURE 2:**
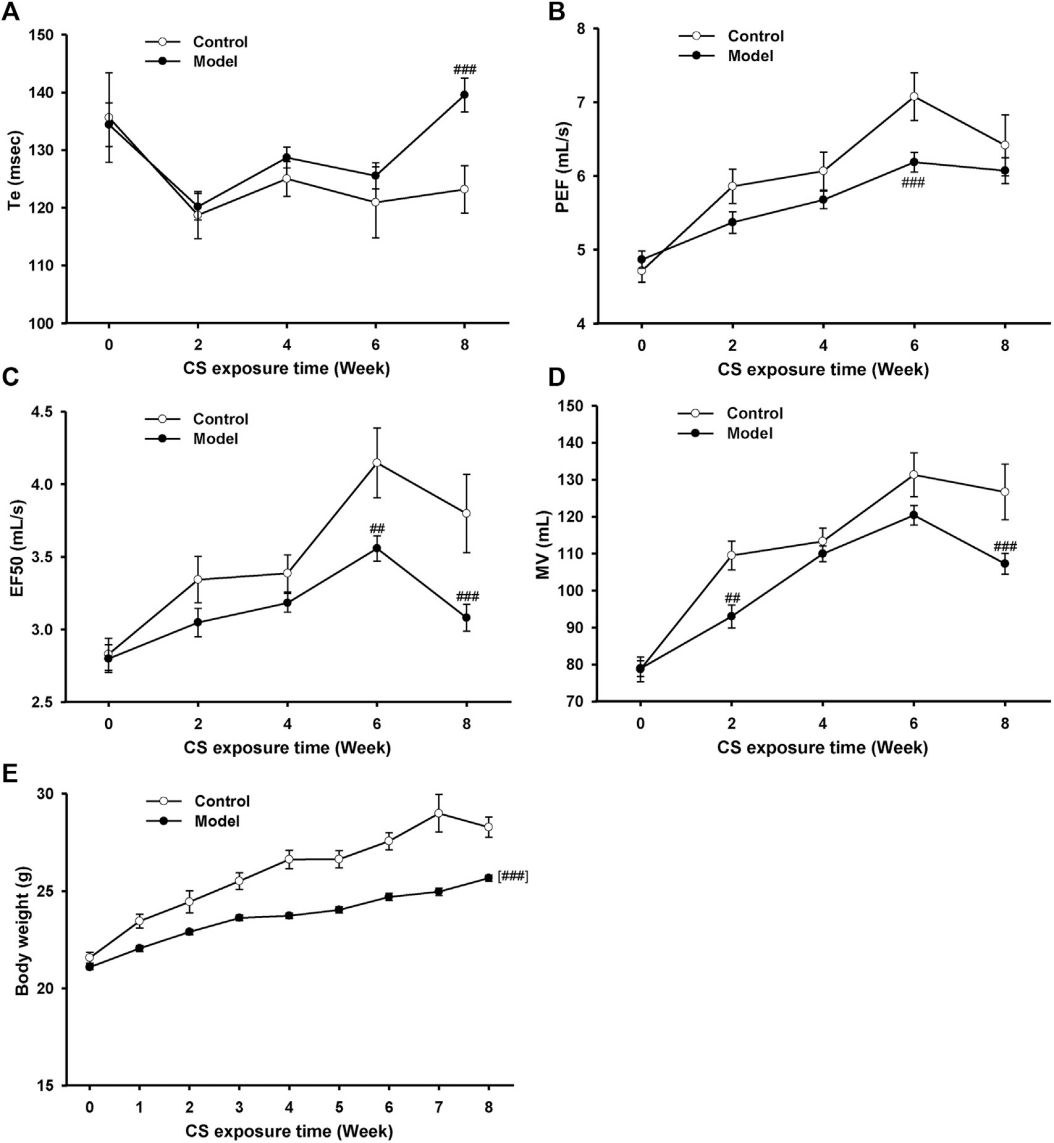
Pulmonary function and body weight changes in mice exposed to cigarette smoke from 0∼8 weeks. **(A)** Expiratory time (Te), **(B)** peak expiratory flow (PEF), **(C)** expiratory flow at 50% tidal volume (EF50), **(D)** maximum minute ventilation (MV), and **(E)** body weight in control and model mice. Data are expressed as means ± SEM (*n* = 12 of control mice, *n* = 60 of model mice). ^##^ *p* < 0.01 and ^###^ *p* < 0.001 vs. control group.

To establish an AECOPD mice model, mice were exposure to CS once daily for 18 weeks and then were intranasally challenged with H1N1 virus, considering that influenza virus infection is one of the major causes of AECOPD (MacLeod et al., 2021). During the first 8 weeks, the lung function in conscious mice was detected biweekly (Figures 2A–D). Compared with the control group, the Te increased while PEF, EF50, and MV decreased in model mice from 6 to 8 weeks, indicating that the pulmonary function declined in model mice after 8 weeks of CS exposure. In addition, a significant decline in body weight was observed in model mice (Figure 2E).

**FIGURE 3:**
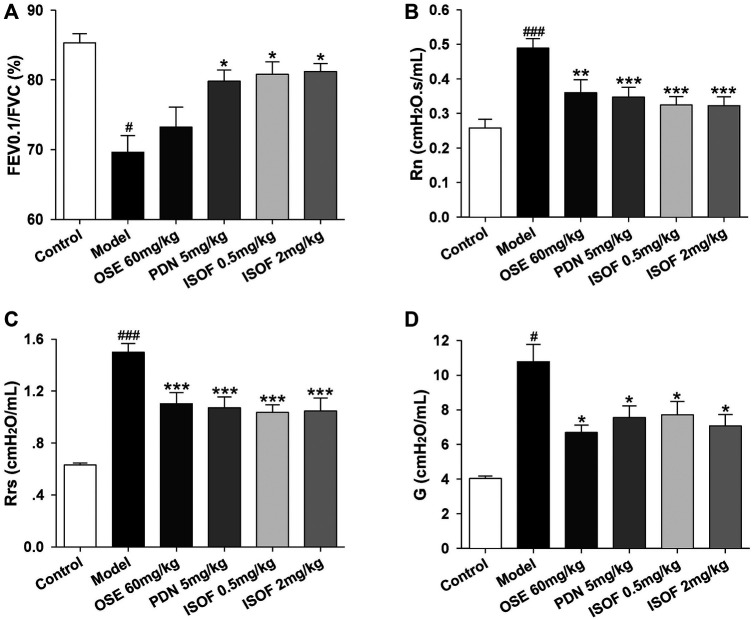
ISOF improved the pulmonary function in AECOPD model mice. **(A)** Forced expiratory volume in 0.1 s/forced vital capacity (FEV0.1/FVC), **(B)** Newtonian resistance (Rn), **(C)** respiratory resistance (Rrs) and **(D)** tissue damping **(G)** in each group. Data are expressed as means ± SEM (*n* = 12 in each group). ^#^ *p* < 0.05 and ^###^ *p* < 0.001 vs. control group; *p < 0.05, **p < 0.01, and ***p < 0.001 vs. model group.

Then, mice were orally treated with ISOF or PDN from week 9. OSE was administered intragastrically before H1N1 infection. Pulmonary function in conscious mice from 9∼17 weeks showed that ISOF and PDN could partly improve lung function in CS-exposed mice (Supplementary Figure 1). Invasive lung function was measured 4 days after infection (Figures 3A–D). The results showed that CS exposure and H1N1 infection decreased the lung function ventilation parameter FEV0.1/FVC% and increased the resistance parameters Rn, Rrs, and G in model mice. Compared with the model group, ISOF (0.5 or 2.0 mg/kg) treatment significantly increased FEV0.1/FVC%, as well as decreased Rn, Rrs, and G. A similar effect was observed for PDN, while OSE had little effect on FEV0.1/FVC%. Thus, both ISOF and PDN could improve the pulmonary ventilation function in AECOPD model mice.

### Isoforskolin Ameliorated Pathological Injury and Reduced Lung Index in Acute Exacerbation of COPD Model Mice

**FIGURE 4:**
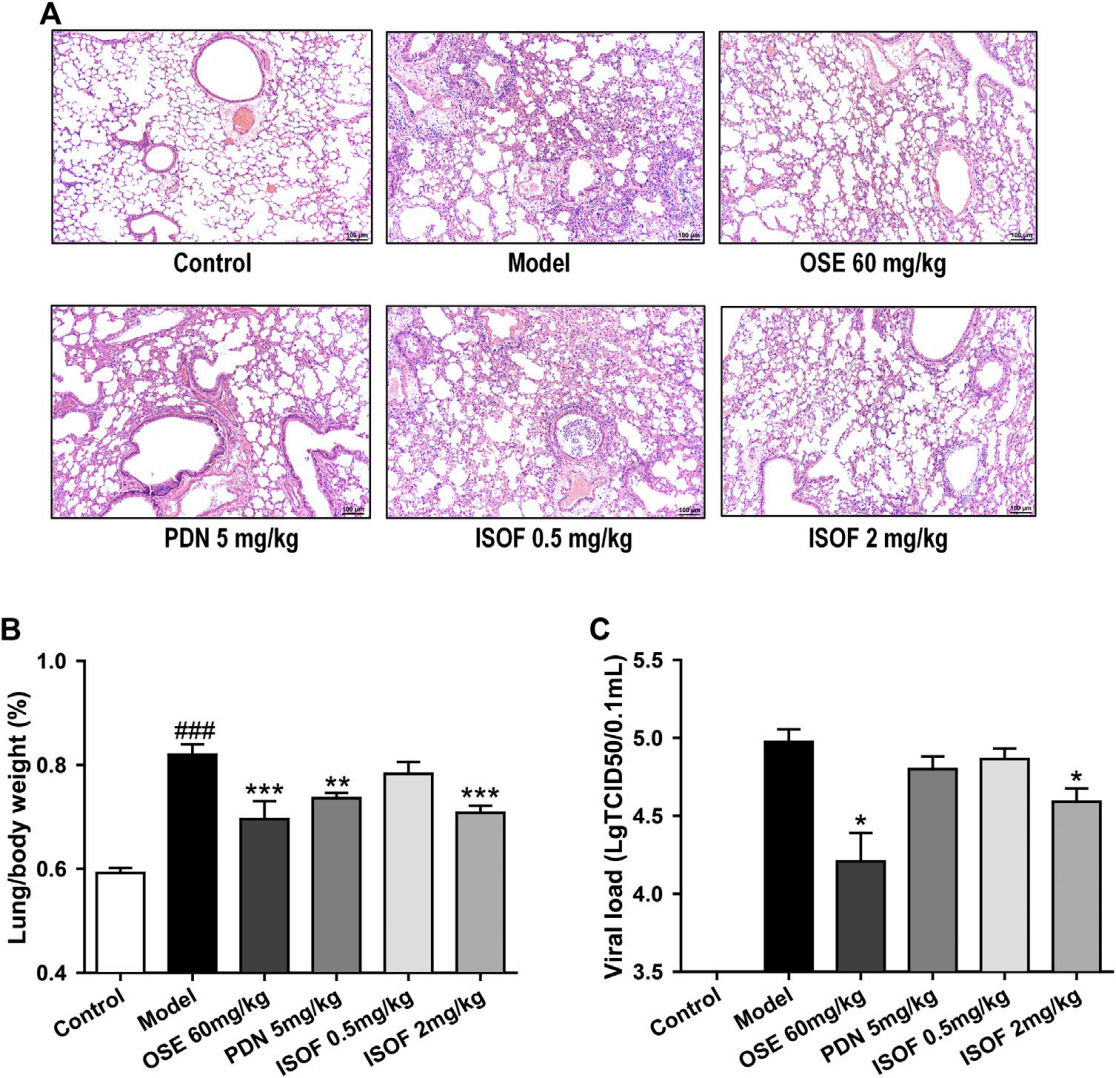
ISOF ameliorated pathological damage of lung tissue in AECOPD model mice. **(A)** Pathological changes in paraffin sections of lung tissue were measured by hematoxylin and eosin (H&E) staining (original magnification: 100×). Scale bar = 100 μm. **(B)** Lung/body weight ratio in each group (*n* = 12). **(C)** Pulmonary viral load in each group (*n* = 6). Data are representative images or expressed as means ± SEM. ^###^ *p* < 0.001 vs. control group; *p < 0.05, **p < 0.01, and ***p < 0.001 vs. model group.

The effect of ISOF on histopathological changes in the lung tissues of AECOPD model mice was evaluated. As shown in Figure 4A, lung sections from CS-exposed and H1N1-infected mice in the model group displayed leukocyte infiltration, alveolar wall thickening, and pulmonary consolidation. Irregular alveolar enlargement, features of pulmonary emphysema, can also be observed in model mice. ISOF (0.5 or 2.0 mg/kg) treatment, as well as PDN and OSE, could improve the alveolar architecture and inhibit the inflammatory infiltration in the bronchial wall and alveolar, with reduced inflammatory score in lung parenchyma (Supplementary Figure 2). The results indicated that treatment with ISOF, PDN, or OSE could relieve the pathological changes in model mice. Meanwhile, the lung index (lung/body weight) of mice in the model group increased apparently (Figure 4B), indicating severe inflammation and pathological injury of the lung tissue. Higher dose of ISOF (2.0 mg/kg), PDN, or OSE treatment reduced the lung index of AECOPD model mice. In addition, a viral load in the lung homogenate was detected (Figure 4C). As expected, OSE significantly reduced the viral titers in the lung homogenate and improved weight loss in AECOPD model mice. ISOF (2.0 mg/kg) treatment could also reduce the viral load in the lung homogenate compared with the model group and partially ameliorate the weight loss in model mice on day 4 after infection (Supplementary Figure 3).

### Isoforskolin Reduced Pulmonary Inflammation in Acute Exacerbation of COPD Model Mice

**FIGURE 5:**
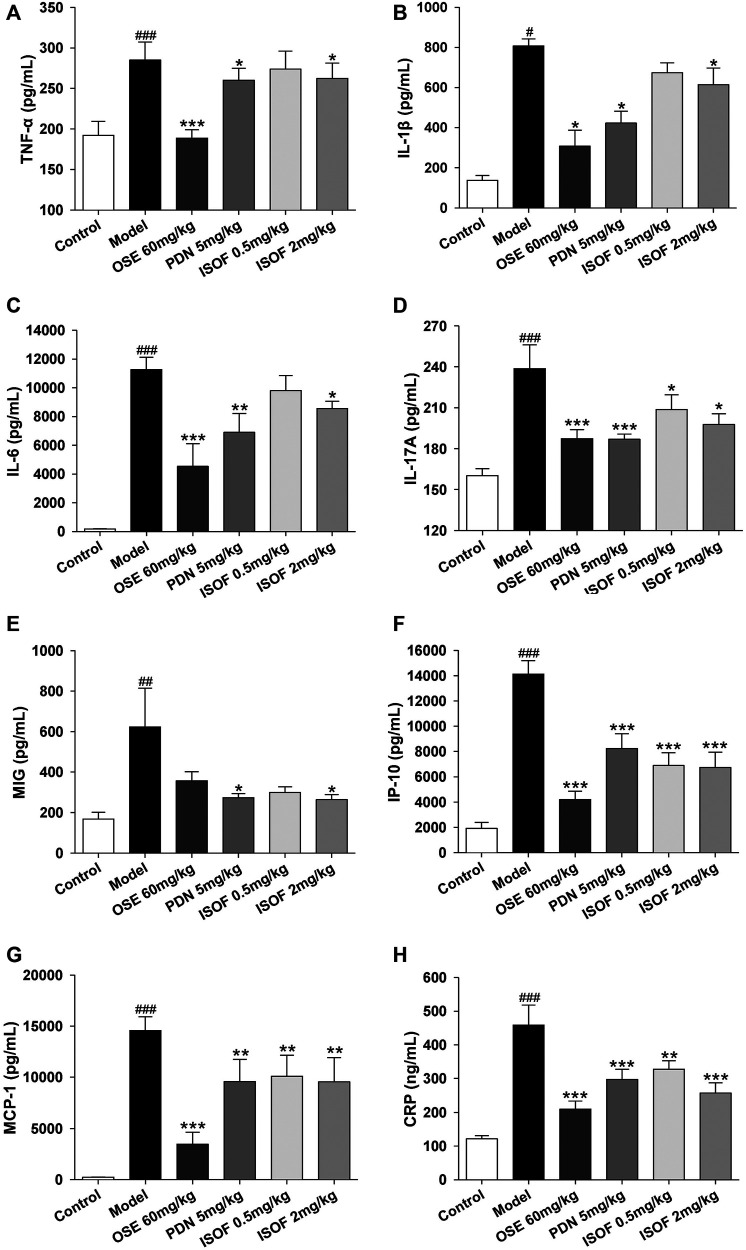
ISOF decreased proinflammatory cytokines in the lung homogenate of AECOPD model mice. The levels of TNF-α **(A)**, IL-1β **(B)**, IL-6 **(C)**, IL-17A **(D)**, MCP-1 **(E)**, MIG **(F)**, IP-10 **(G),** and CRP **(H)** in each group were measured by the Bio-Plex Pro Mouse Cytokine assay or ELISA. Data are expressed as means ± SEM (*n* = 6 in each group). ^#^ *p* < 0.05, ^##^ *p* < 0.01, and ^###^ *p* < 0.001 vs. control group; *p < 0.05, **p < 0.01, and ***p < 0.001 vs. model group.

In order to assess the effect of ISOF on pulmonary inflammation in AECOPD model mice, proinflammatory cytokines TNF-α, IL-1β, IL-6, IL-17A, MCP-1, MIG, IP-10, and CRP in the lung homogenate were detected. As shown in Figures 5A–H, the levels of these inflammatory cytokines were increased dramatically in CS-exposed and H1N1-infected mice. Both PDN and OSE could potently inhibit the expression of inflammatory cytokines in lung tissues. Also, ISOF treatment effectively decreased the levels of inflammatory cytokines in model mice, with better effect at high dose. The results indicated that ISOF could attenuate the inflammatory reaction of lung tissues in AECOPD model mice.

### Isoforskolin Reduced Th17 Cells and Related Protein Levels in Acute Exacerbation of COPD Model Mice

**FIGURE 6:**
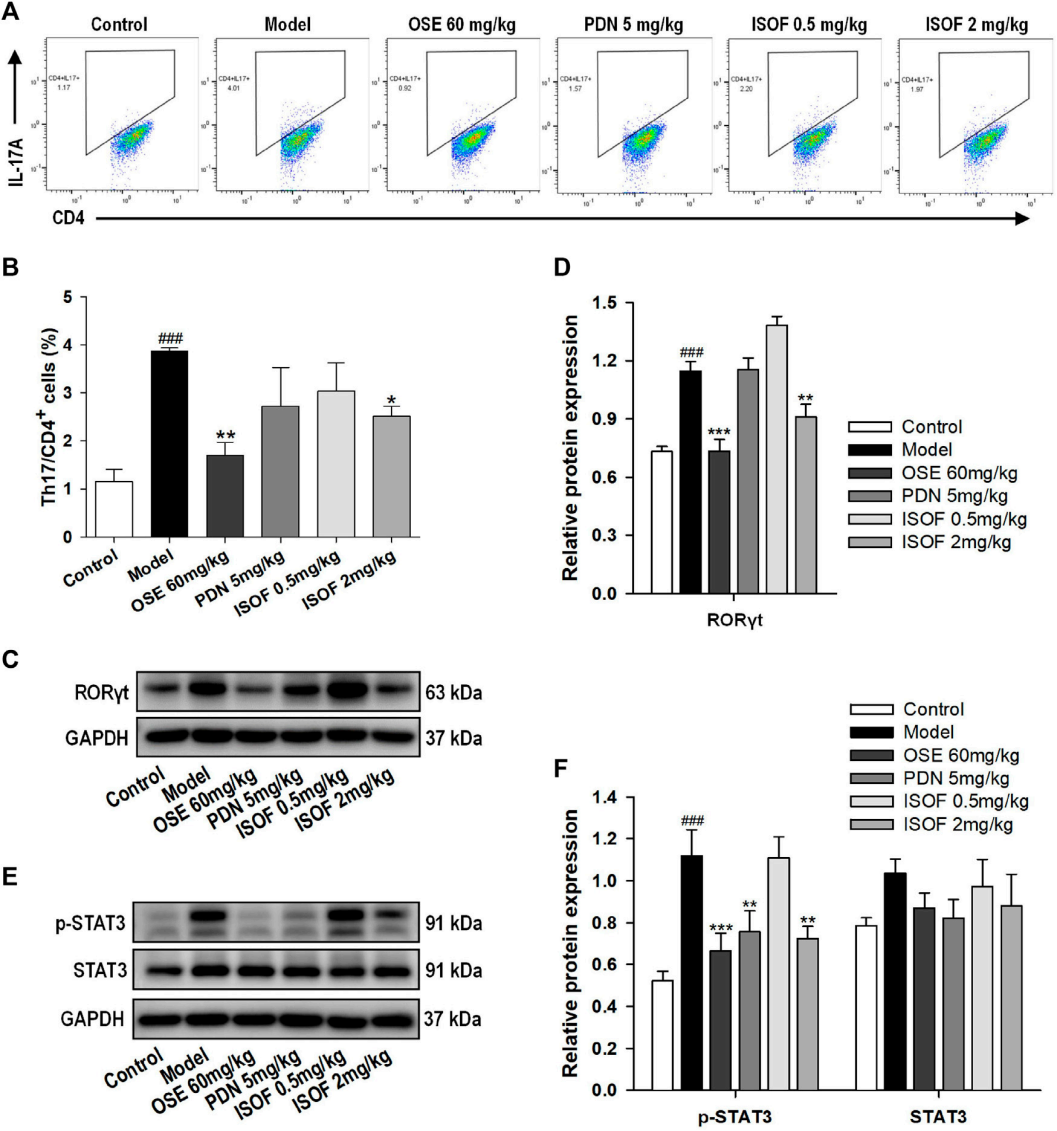
ISOF reduced Th17 cells and the protein levels of RORγt and p-STAT3 in the lung tissues of AECOPD model mice. **(A)** Representative flow cytometric dot-plots of Th17 cells in the lung tissues of each group (Th17: CD4^+^IL-17A^+^ cells). **(B)** The proportion of Th17 cells was analyzed. **(C)** The protein levels of RORγt in the lung tissues of each group were assessed by western blot. **(D)** The relative density of RORγt to GAPDH in each group. **(E)** The protein levels of STAT3 and p-STAT3 in the lung tissues of each group were assessed by western blot. **(F)** The relative density of STAT3 or p-STAT3 to GAPDH in each group. Data are expressed as means ± SEM (*n* = 4 in each group). ^###^ *p* < 0.001 vs. control group; *p < 0.05, **p < 0.01, and ***p < 0.001 vs. model group.

Th17 cells and related cytokines play an important role in pulmonary inflammation (Holloway and Donnelly, 2013; Phillips et al., 2015). To further evaluate the effect of ISOF on Th17 cells in the AECOPD model, flow cytometry was used to detect the Th17 cells in lung tissues. Representative flow cytometric dot-plots are shown in Figure 6A. Compared with the control group, the proportion of Th17 cells (CD4^+^IL-17A^+^ T cells) was significantly increased in the model group (Figure 6B). ISOF (2.0 mg/kg) or OSE treatment decreased the percentage of Th17 cells. The development of Th17 cells is dependent on the signal transducer and activator of transcription 3 (STAT3) and retinoid-related orphan receptor RORγt, which is a Th17-specific transcription factor (Dong, 2008). Thus, the protein expression of RORγt, p-STAT3, and STAT3 in lung tissues was examined (Figures 6C–F). Compared with the control group, the protein levels of RORγt and p-STAT3 were increased significantly in lung tissues of AECOPD model mice. Both ISOF (2.0 mg/kg) and OSE were able to downregulate the expression of RORγt and the phosphorylation level of STAT3. Collectively, these results indicated that ISOF might regulate the Th17/IL-17A axis in the lung tissues of AECOPD model mice.

### Isoforskolin Downregulated NF-κB Signaling in Acute Exacerbation of COPD Model Mice

**FIGURE 7:**
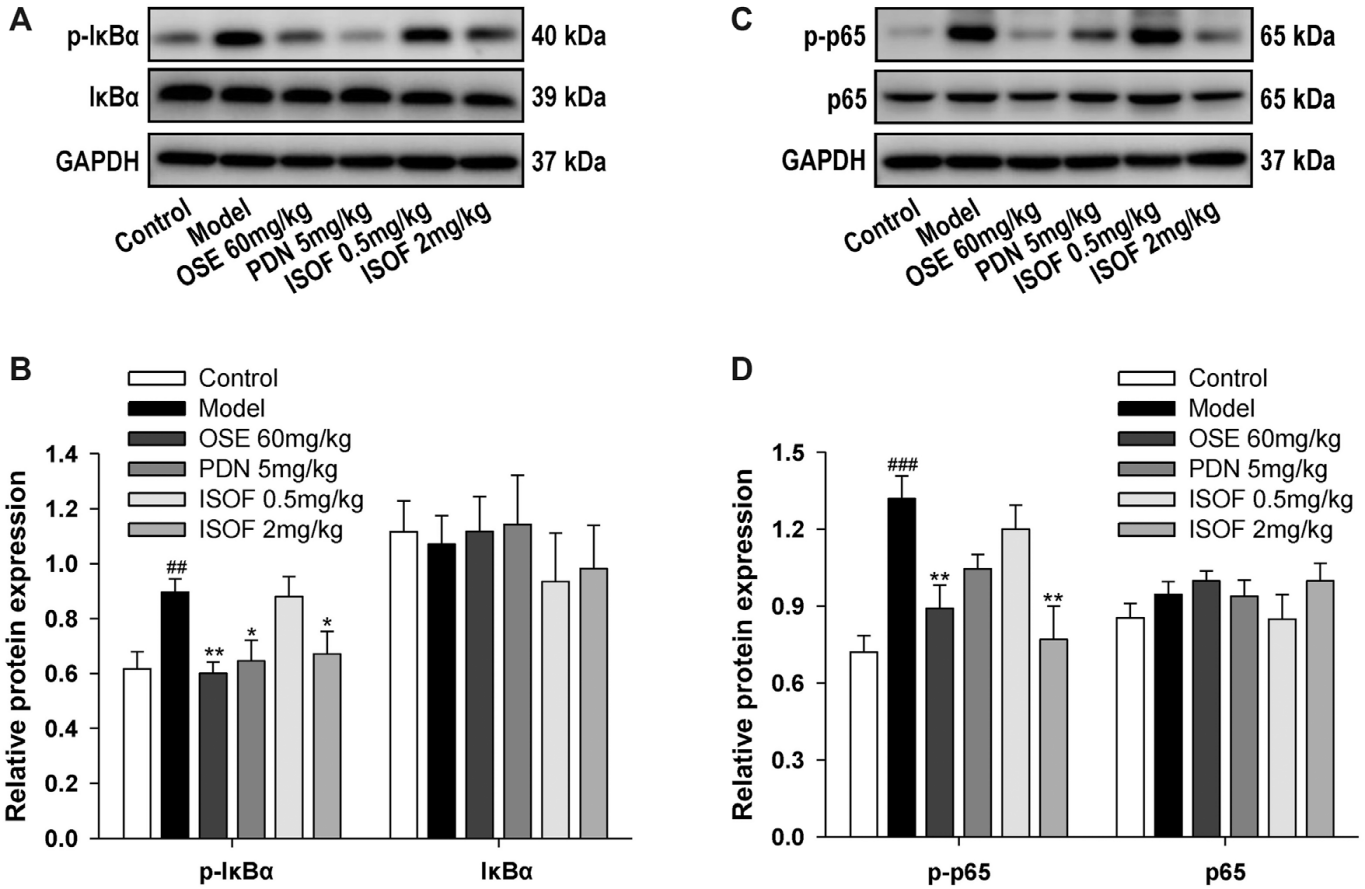
ISOF downregulated NF-κB signaling pathway activity in the lung tissues of AECOPD model mice. **(A)** The protein levels of IκB and p-IκB in the lung tissues of each group were assessed by western blot. **(B)** The relative density of IκB or p-IκB to GAPDH in each group. **(C)** The protein levels of NF-κB and p-NF-κB in the lung tissues of each group were assessed by western blot. **(D)** The relative density of NF-κB or p-NF-κB to GAPDH in each group. Data are expressed as means ± SEM (*n* = 4 in each group). ^##^ *p* < 0.01 and ^###^ *p* < 0.001 vs. control group; *p < 0.05 and **p < 0.01 vs. model group.

NF-κB signaling pathway is a critical participant in the pathogenesis of COPD by regulating the expression of inflammatory mediators (Schuliga, 2015). Normally, NF-κB p50 and p65 dimers are bound to IκB and stay inactive in the cytoplasm (Gerlo et al., 2011). The phosphorylation and ubiquitination of IκB will lead to the release and translocation of NF-κB dimers (Gerlo et al., 2011). Therefore, the protein expression of p-IκB, IκB, p-p65, and p65 in lung tissues was detected (Figures 7A–D). The results showed that levels of p-IκB and p-p65 were increased markedly in the model group, while ISOF, PDN, or OSE treatment significantly reduced the phosphorylation level of IκB and NF-κB p65. The results suggested that ISOF could inhibit the NF-κB signaling pathway in the lung tissues of AECOPD model mice.

### Isoforskolin Attenuated NLRP3 Inflammasome Activation in Acute Exacerbation of COPD Model Mice

**FIGURE 8:**
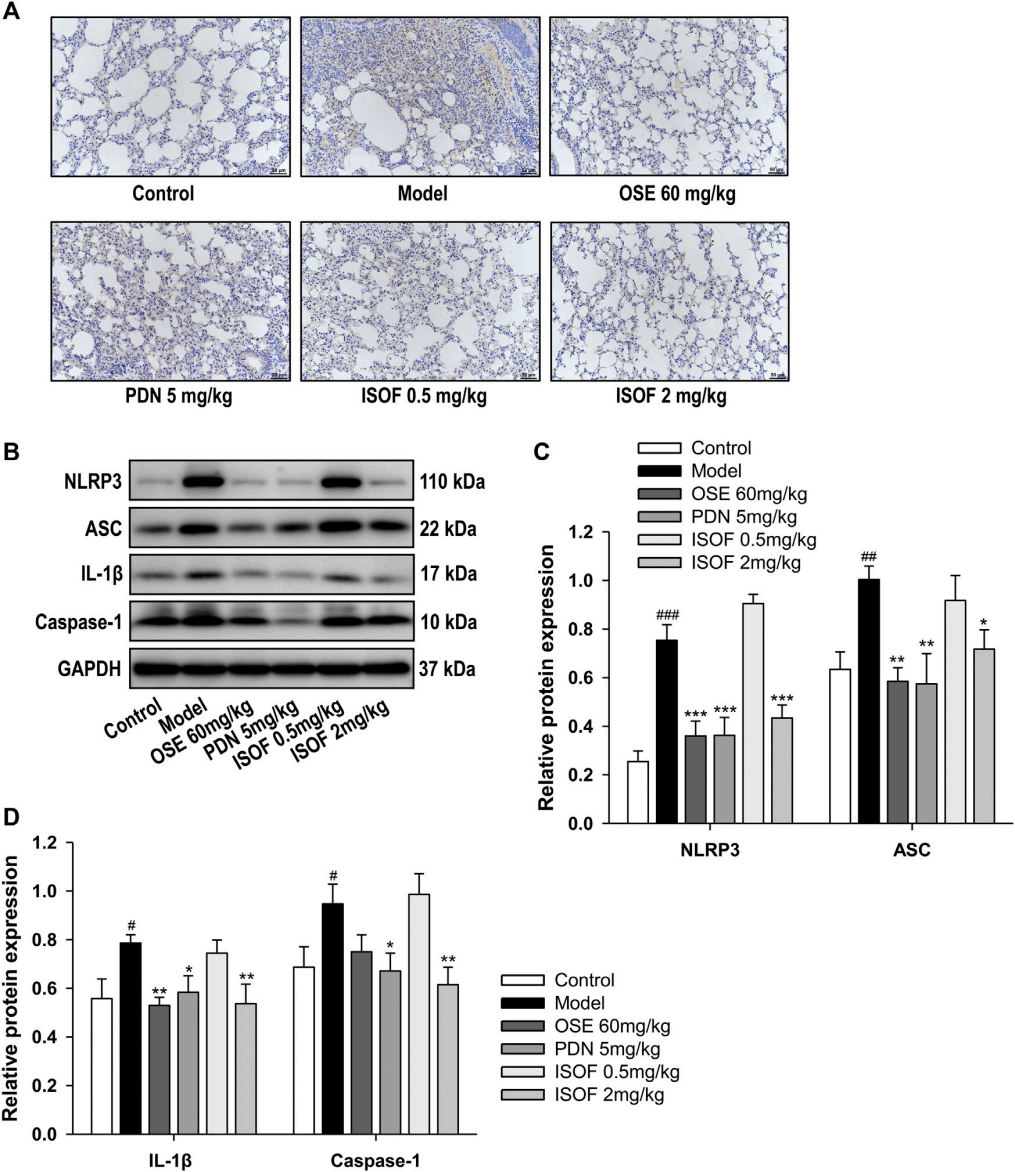
ISOF attenuated NLRP3 inflammasome activation in the lung tissues of AECOPD model mice. **(A)** Immunohistochemistry analysis of NLRP3 expression in the lung tissues of each group (original magnification: 200×). Scale bar = 50 μm. **(B)** The protein levels of NLRP3, ASC, IL-1β, and caspase-1 in the lung tissues of each group were assessed by western blot. **(C)** The relative density of NLRP3 or ASC to GAPDH in each group. **(D)** The relative density of IL-1β or caspase-1 to GAPDH in each group. Data are expressed as means ± SEM (*n* = 4 in each group). ^#^ *p* < 0.05, ^##^ *p* < 0.01, and ^###^ *p* < 0.001 vs. control group; *p < 0.05, **p < 0.01, and ***p < 0.001 vs. model group.

Inflammasome activation is involved in COPD pathogenesis, since the increased inflammasome activation and IL-1β level in COPD patients has been reported (Kim et al., 2015). To explore the effect of ISOF on NLRP3 inflammasome activation in AECOPD model mice, we further detected the protein expression of NLRP3, ASC, caspase-1, and IL-1β in the lung tissues. Immunohistochemistry analysis showed obvious NLRP3 expression in the lung tissues of the model group, with much lower expression in the control group and other treatment groups (Figure 8A). Western blot analysis showed that the protein expression levels of NLRP3, ASC, caspase-1, and IL-1β were increased significantly in the model group and treatment with ISOF, PDN, or OSE downregulated the expression of these proteins (Figures 8B–D). Therefore, ISOF could attenuate NLRP3 inflammasome activation in the lung tissues of AECOPD model mice.

## Discussion

Acute exacerbation, associated with increased airway inflammation and mucus production, is the main reason of poor outcomes and mortality in COPD (Whittaker Brown and Braman, 2020; MacLeod et al., 2021; Waeijen-Smit et al., 2021). Currently, the therapeutic drugs of COPD mainly focus on reliving symptoms, and finding new therapies to prevent the disease progression is still challenging (Jarnicki et al., 2016; Wang et al., 2020). Emerging evidence suggests that increasing intracellular cAMP level is a promising strategy to reduce inflammation and immunomodulation in COPD (Raker et al., 2016; Schmidt et al., 2020; Wang et al., 2020). Thus, in this study, we hypothesized that ISOF, an AC agonist, could attenuate AECOPD through anti-inflammation and immune regulation.

Establishing an animal model to mimic the features of COPD could improve the research on pathophysiology and treatment of this disease (Ghorani et al., 2017). A previous study has established a mice model with exacerbation of pulmonary inflammation induced by CS exposure and H1N1 infection, in which mice were exposed to CS only for 10 days (Bucher H et al., 2016). Since long-term CS exposure would better mimic the development and progression of COPD (Phillips et al., 2015; Ghorani et al., 2017), we established an AECOPD mice model by CS exposure for 18 weeks and subsequently H1N1 virus infection. This AECOPD mice model is characterized by significantly decreased lung function, severe pathological injury, and increased lung index and proinflammatory cytokines, indicating that it is a valuable AECOPD mice model suitable for further investigation.

Previous studies have shown that ISOF could attenuate inflammatory responses in different cells or animal models (Yang et al., 2011; Liang et al., 2017; Zhao et al., 2017; Du et al., 2019). In our study, we demonstrated for the first time that ISOF inhibited inflammatory injury and downregulated proinflammatory cytokine levels in the lung tissues of AECOPD model mice. More importantly, ISOF could improve the pulmonary function in AECOPD model mice with increased ventilation parameters and decreased resistance parameters. It has been reported that ISOF can relax the isolated guinea pig trachea induced by histamine or acetylcholine (Wang et al., 2013). Thus, the anti-inflammation and tracheal relaxation effects of ISOF might contribute to the improved lung function in AECOPD model mice. As an AC agonist, ISOF showed potent therapeutic efficacy on AECOPD model mice, indicating that activating AC might be a promising strategy for the prevention and treatment of AECOPD.

PDN and OSE were used as positive drugs in the study, considering that systemic corticosteroids are efficacious for AECOPD treatment in clinic and OSE is effective in influenza virus–infected COPD patients (Li et al., 2020; Whittaker Brown and Braman, 2020). As a powerful anti-inflammatory drug, PDN significantly ameliorated the key features in AECOPD model mice. OSE could reduce the viral load, suppress proinflammatory cytokine production, and decrease resistance parameters of lung function in AECOPD model mice. Since the airway inflammatory responses would lead to worsening of airflow limitation during COPD exacerbations (Wedzicha and Seemungal, 2007), the improved lung function parameters of PDN and OSE might be attributed to the reduced inflammatory responses in AECOPD model mice.

Accumulating evidence has demonstrated that NF-κB plays a pivotal role in the inflammatory responses and NLRP3 inflammasome activation in multiple inflammatory diseases (Schuliga, 2015; Liu et al., 2017). Bacterial or viral infection activates NF-κB through toll-like receptors, resulting in the production of proinflammatory cytokines including TNF-α, IL-1β, and IL-6 (Liu et al., 2017). NF-κB also serves as the priming signal to facilitate the transcriptional expression of NLRP3 and pro-IL-1β (Liu et al., 2017; Zhao and Zhao, 2020). The activation of NLRP3 inflammasome participates in the pathogenesis of AECOPD by the secretion of IL-1β and IL-18, activating immune cells to trigger airway inflammation (Faner et al., 2016; Hikichi et al., 2019). Previously, we have reported that ISOF and forskolin attenuated LPS-induced inflammation in human mononuclear leukocytes through TLR4/MyD88/NF-κB cascades (Du et al., 2019). Also, it has been reported that forskolin could attenuate the activation of NLRP3 inflammasome in human macrophages (Chen et al., 2019). In the AECOPD model mice, we found that ISOF significantly downregulated the phosphorylation level of IκB and NF-κB p65, as well as the protein levels of NLRP3, ASC, caspase-1, and IL-1β, leading to the reduced proinflammatory cytokines in the lung tissues. Thus, ISOF alleviated inflammatory responses in AECOPD model mice partially through the inhibition of NF-κB/NLRP3 pathway, which might be related to the AC activation by ISOF, given that increasing the intracellular level of cAMP could inhibit NF-κB function in the presence of proinflammatory stimuli (Gerlo et al., 2011). In addition, PDN and OSE also showed potent inhibition of NF-κB/NLRP3 pathway, contributing to their therapeutic efficiency in AECOPD model mice.

Th17 cell and related cytokines are involved in COPD pathogenesis and exacerbations (Le Rouzic et al., 2017). The activation of NF-κB/NLRP3 pathway would promote the adaptive immune responses, in which Th17 cells play an important role (Liu et al., 2017; Evavold and Kagan, 2018). Proinflammatory cytokine IL-6 together with low concentrations of transforming growth factor β (TGF-β) can induce the differentiation of Th17 cells (Torchinsky and Blander, 2010), and IL-1β and IL-23 are also required for the stable differentiation of Th17 cells (Torchinsky and Blander, 2010; Evavold and Kagan, 2018). Cytokines IL-6 and IL-23 can activate STAT3, which induces the expression of RORγt, leading to the secretion of IL-17A (Dong, 2008; Torchinsky and Blander, 2010). IL-17A can promote the activation of bronchial fibroblasts and epithelial cells to produce proinflammatory cytokines and recruit neutrophils and macrophages to sites of inflammation, aggravating the COPD progress (Halwani et al., 2013; Ponce-Gallegos et al., 2017). Our results showed that ISOF decreased the protein expression of p-STAT3 and RORγt, as well as Th17 cells and IL-17A level in the lung tissues of AECOPD model mice, indicating that ISOF could modulate the Th17/IL-17A axis to reduce inflammatory responses in AECOPD model mice.

**FIGURE 9:**
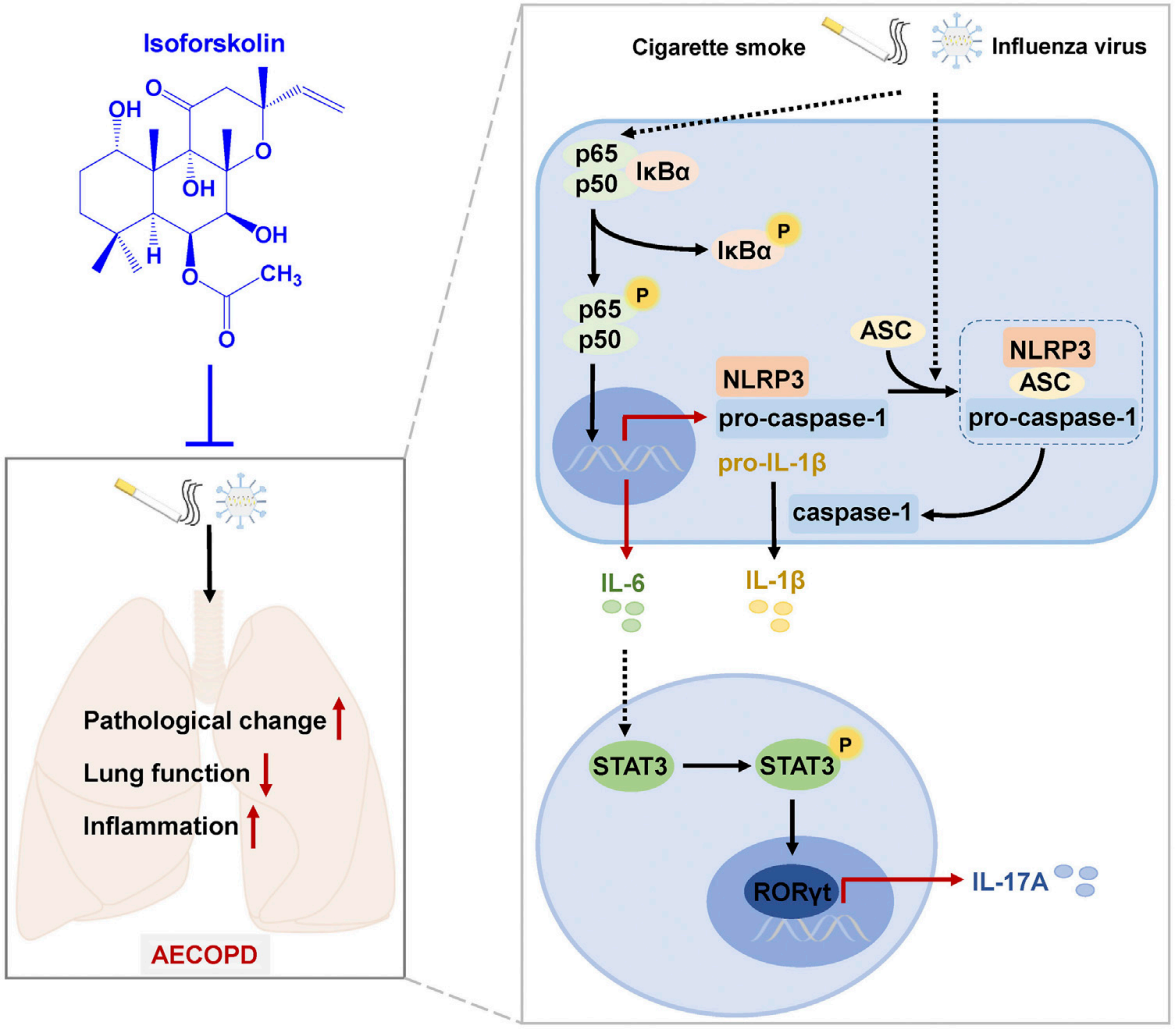
A schematic diagram of the potential mechanisms by which ISOF treatment alleviates AECOPD *in vivo*. AECOPD model mice induced by cigarette smoke exposure and H1N1 virus infection have decreased lung function and increased pathological injury and inflammation, as well as activated NF-κB signaling and NLRP3 inflammasome and increased Th17 cells and IL-17A level in lung tissues. ISOF treatment attenuates the phosphorylation of NF-κB and the activation of NLRP3 inflammasome and decreases Th17 cells and IL-17A level in lung tissues, which could contribute to the decreased inflammatory response and improved lung function in AECOPD model mice. Thus, ISOF is a promising candidate for the prevention and treatment of AECOPD.

In summary, we have established an AECOPD mice model by CS exposure and H1N1 virus infection, which had decreased lung function and increased pathological injury and inflammation (Figure 9). ISOF treatment significantly alleviated AECOPD by improving lung ventilation function, downregulating proinflammatory cytokine level and attenuating pathological injury. The underlying mechanism is shown in Figure 9. CS exposure and H1N1 virus infection can activate NF-κB signaling pathway and NLRP3 inflammasome and promote proinflammatory cytokine (IL-6, IL-1β) release and Th17 cell differentiation. ISOF treatment inhibited the phosphorylation of IκB and NF-κB p65 and downregulated the protein levels of NLRP3, ASC, caspase-1, and IL-1β. Also subsequently, ISOF decreased the phosphorylation of STAT3 and the expression of RORγt to inhibit Th17 differentiation and IL-17A secretion.

## Conclusion

In conclusion, our results suggest that ISOF can effectively improve pulmonary function and alleviate pathological injury and inflammation in AECOPD model mice induced by CS exposure and H1N1 virus infection. Mechanistically, ISOF significantly decreased Th17 cells and IL-17A level and downregulated the NF-κB/NLRP3 signaling pathways in the lung tissues of AECOPD model mice. Our findings support ISOF as a promising candidate for the prevention and treatment of AECOPD.

## Data Availability

The original contributions presented in the study are included in the article/Supplementary Material; further inquiries can be directed to the corresponding authors.
