# Supplementary material for: Isoforskolin Alleviates AECOPD by Improving Pulmonary Function and Attenuating Inflammation Which Involves Downregulation of Th17/IL-17A and NF-κB/NLRP3
Source: Front Pharmacol. 2021 Jul 30;12:721273. doi: 10.3389/fphar.2021.721273 (PMC8361481; doi:10.3389/fphar.2021.721273)
Supplement: Supplementary file 1 [file DataSheet1.pdf]

Supplementary Figures

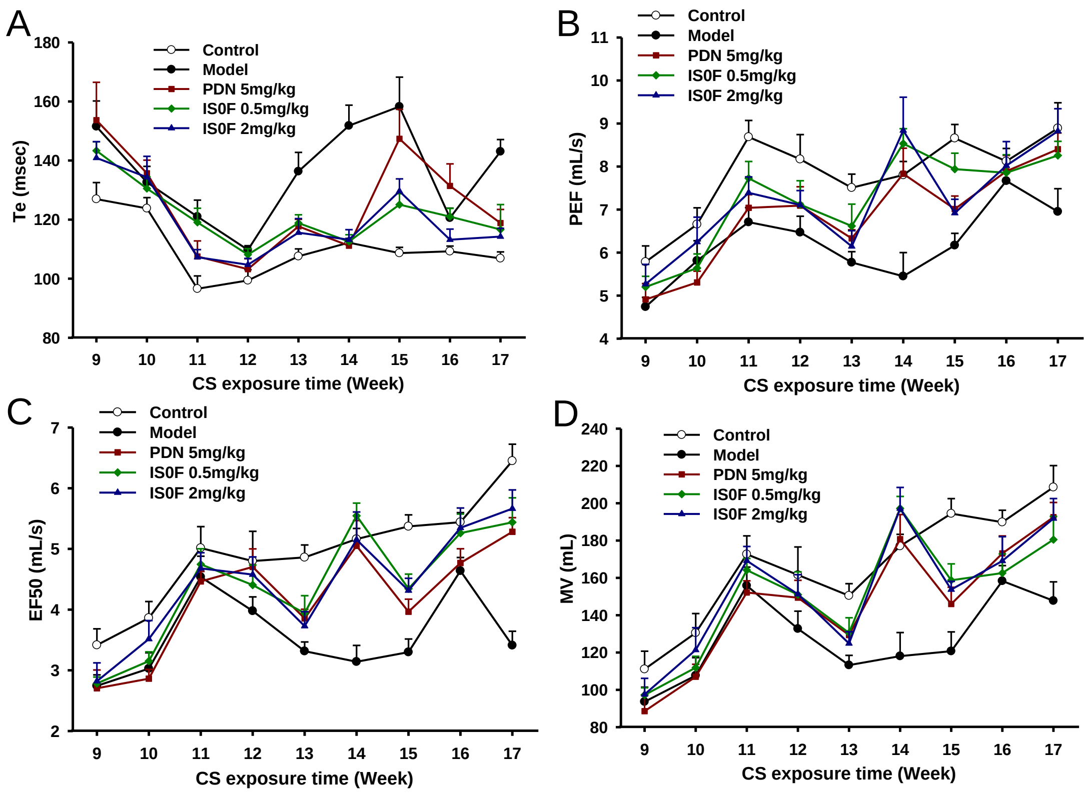

FIGURE 1 Pulmonary function in conscious mice from 9~17 weeks in each group. **(A)** Expiratory time (Te), **(B)** Peak expiratory flow (PEF), **(C)** Expiratory flow at 50 % tidal volume (EF50), and **(D)** Maximum minute ventilation (MV) in each group. Data are expressed as means ± SEM (n = 12 in each group).

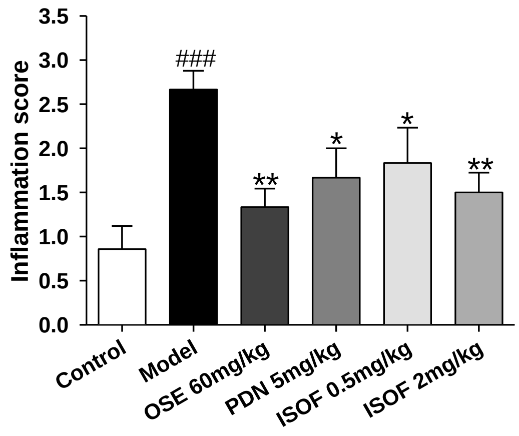

FIGURE 2 Inflammation score of lung tissue by H&E staining in each group. Data are expressed as means ± SEM (n = 6 in each group). $^{###}P < 0.001$ versus control group; $^{*}P < 0.05$, $^{**}P < 0.01$ versus model group.

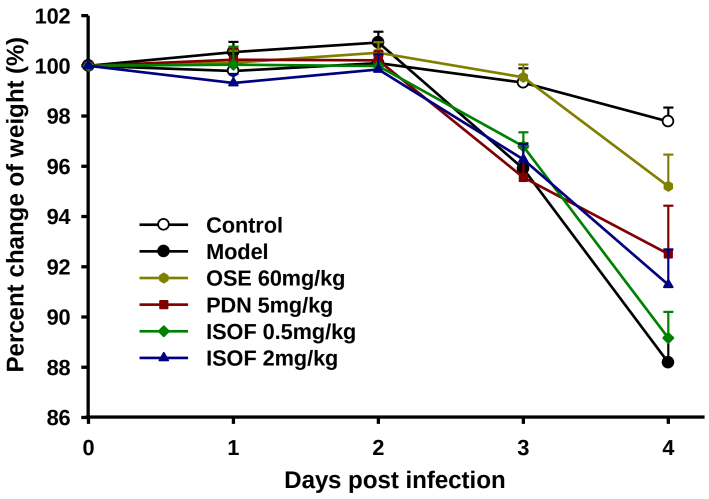

FIGURE 3 Body weight changes in each group after H1N1 virus infection. Data are expressed as means ± SEM (n = 12 in each group).
